# Are giant Brunner’s gland hyperplasia and gastric heterotopia unknown complications of Roux-en-Y gastric bypass?

**DOI:** 10.1055/a-2134-9501

**Published:** 2023-08-21

**Authors:** Clara Yzet, Pierre Lafeuille, Elise Pelascini, Jérôme Rivory, Valérie Hervieu, Mathieu Pioche

**Affiliations:** 1 Gastroenterology and Endoscopy Unit, Pavillon L, Edouard Herriot Hospital, Lyon, France; 2 Department of Gastrointestinal Surgery, Edouard Herriot Hospital, Lyon, France; 3 Department of Pathology, Edouard Herriot Hospital, Lyon, France; 4 Inserm U1032, Labtau, Lyon, France

The exploration of the excluded duodenum is challenging after a Roux-en-Y gastric bypass (RYGB). Endoscopic ultrasound (EUS)-directed endoscopic retrograde cholangiopancreatography (EDGE) is an emerging endoscopic method that allows examination of the duodenum after placement of an apposition stent 1 .

We report the cases of three patients who were previously treated by RYGB for severe obesity. The patients were subsequently referred following the discovery of duodenal lesions > 1.5 cm in size ( Fig. 1 ) on systematic computed tomography scan performed for various nonspecific symptoms.

**Fig. 1:**
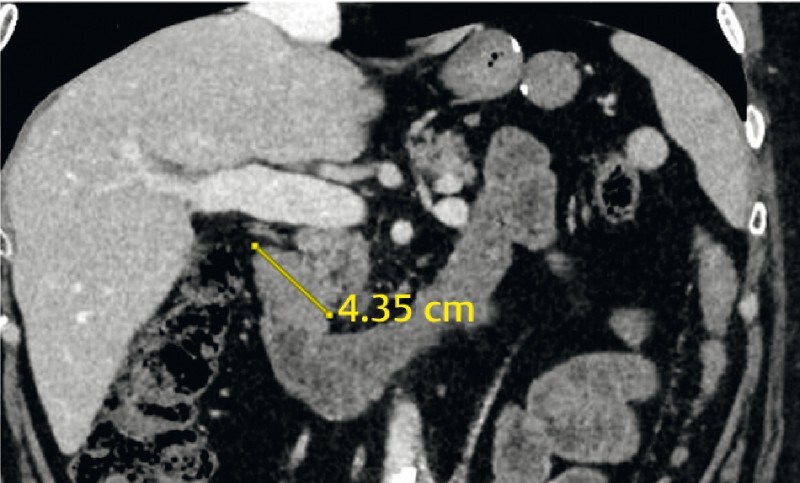
Computed tomography scan showing a 4.3-cm lesion in the first duodenum.

To examine the lesion, an EDGE procedure was performed. First, a gastro-gastrostomy was created to access the bypassed stomach ( Video 1 ). After locating the excluded stomach, under EUS guidance a lumen-apposing metal stent (LAMS) was deployed to reconnect the excluded stomach. The exploration of the duodenum was performed immediately for two patients, who benefited from a one-step procedure using a transnasal scope to pass through the LAMS and perform the resection using a small-caliber snare by underwater technique. The third patient underwent resection with a conventional scope 1 week later.

**Video 1** The potential for development of giant Brunner’s gland hyperplasia and gastric heterotopia after Roux-en-Y gastric bypass.


In all cases, the duodenal lesion harbored a hyperplastic regular pattern. Analysis of the polyp confirmed the presence of gastric heterotopia in two patients and a Brunner’s gland hyperplasia without dysplasia in one patient. Gastric heterotopia is a benign condition with a reported incidence of 0.5 %–14 % 2 . Gastric acid has been suggested to participate in the development of gastric heterotopia. Brunner’s gland hyperplasia is a rare duodenal lesion; it usually occurs in response to an acidic environment or *Helicobacter pylori* infection. These three consecutive cases raise the question of the potential risk of developing such lesions due to excess acid after gastric bypass. Surgeons and gastroenterologists should be aware of this potential condition before any surgery for duodenal lesions after an RYGB to avoid unnecessary surgery for benign conditions.

Endoscopy_UCTN_Code_CCL_1AB_2AD_3AC

## Notes

Endoscopy E-Videos

https://eref.thieme.de/e-videos
*E-Videos* is an open access online section of the journal *Endoscopy* , reporting on interesting cases and new techniques in gastroenterological endoscopy. All papers include a high-quality video and are published with a Creative Commons CC-BY license. Endoscopy E-Videos qualify for HINARI discounts and waivers and eligibility is automatically checked during the submission process. We grant 100% waivers to articles whose corresponding authors are based in Group A countries and 50% waivers to those who are based in Group B countries as classified by Research4Life (see: https://www.research4life.org/access/eligibility/ ). This section has its own submission website at https://mc.manuscriptcentral.com/e-videos
